# Proteome dataset of pre-ovulatory follicular fluids from less fertile dairy cows

**DOI:** 10.1016/j.dib.2016.04.051

**Published:** 2016-04-26

**Authors:** Maya Zachut, Pankaj Sood, Lilya Livshitz, Gitit Kra, Yishai Levin, Uzi Moallem

**Affiliations:** a Department of Ruminant Science, Institute of Animal Sciences, The Volcani Center, PO Box 6, Bet-Dagan 50250, Israel; b Department of Veterinary Gynecology and Obstetrics, DGCN College of Veterinary and Animal Sciences, Himachal Pradesh Agricultural University, Palampur, Himachal Pradesh 176062, India; c de Botton Institute for Protein Profiling, The Nancy and Stephen Grand Israel National Center for Personalized Medicine, Weizmann Institute of Science, Rehovot, Israel

**Keywords:** Proteomics, Follicular fluid, Reproductive disorders, Cow

## Abstract

This article contains raw and processed data related to research published in Zachut et al. (2016) [1]. Proteomics data from preovulatory follicles in cows was obtained by liquid chromatography-mass spectrometry following protein extraction. Differential expression between controls and less fertile cows (LFC) was quantified using MS1 intensity based label-free. The only previous proteomic analysis of bovine FF detected merely 40 proteins in follicular cysts obtained from the slaughterhouse (Maniwa et al., 2005) [2], and the abundance of proteins in the bovine preovulatory FF remains unknown. Therefore, the objectives were to establish the first dataset of FF proteome in preovulatory follicles of cows, and to examine differentially expressed proteins in FF obtained in-vivo from preovulatory follicles of less fertile cows (also termed “repeat breeder”) and control (CTL) cows. The proteome of FF from 10 preovulatory follicles that were aspirated in vivo (estradiol/progesterone>1) was analyzed. This novel dataset contains 219 identified and quantified proteins in FF, consisting mainly of binding proteins, proteases, receptor ligands, enzymes and transporters. In addition, differential abundance of 8 proteins relevant to follicular function was found in LFC compared to CTL; these findings are discussed in our recent research article Zachut et al. (2016) [1]. The present dataset of bovine FF proteome can be used as a reference for any study involving disorders of follicular development in dairy cows or in comparative studies between species.

**Specifications Table**

**Table:** 

Subject area	*Biology*
More specific subject area	*Reproductive proteomics*
Type of data	*Table*
How data was acquired	*Liquid Chromatography-Mass spectrometry: nanoAcquity+Q Exactive Plus*
Data format	*Raw, identifications, analyzed*
Experimental factors	*Follicular fluid from preovulatory follicles of control and less fertile cows were aspirated in vivo and subjected to proteomic analysis.*
Experimental features	*Ovarian follicles* ≥7 *mm in diameter were aspirated in vivo. The FF from preovulatory follicles (estradiol/progesterone >1) of 5 control and 5 less fertile cows was analyzed by Liquid Chromatography-Mass spectrometry following protein extraction. Differential expression was quantified using MS1 intensity based label-free.*
Data source location	*Bet Dagan, Israel*
Data accessibility	*Data is with this article*

**Value of the data**

• This work provides the first documentation of proteome dataset from pre-ovulatory follicles in cows; 219 proteins were identified and quantified in FF.
• The proteome dataset from bovine FF can be used as a reference for any study involving disorders of the developing or preovulatory ovarian follicles in dairy cows.
• Differential abundance of 8 proteins relevant to follicle function was demonstrated in less fertile cows compared to controls. Further research can be done to explore the characteristics and functioning of these proteins in FF of cows with reproductive disorders.
• This dataset could be used as a benchmark for FF proteomics comparative studies between species.

## 1 Data

This data describes the proteome of follicular fluids from preovulatory follicles aspirated in vivo from less fertile and control dairy cows. Previous analysis of bovine FF detected merely 40 proteins in follicular cysts (Maniwa et al., 2005) [2]). Supplementary Table 1 contains the dataset of 219 identified and quantified proteins obtained by proteomic analysis, as well as statistical analysis of differentially abundant proteins between LFC and control cows.

## 2 Experimental design, materials and methods

### 2.1 Animals and procedures

The procedures used were approved by the Volcani Center Animal Care Committee. Eighteen Israeli Holstein lactating cows (>60 d in lactation) at the Volcani Center Experimental Farm (Bet Dagan, Israel) were used. Details on cows and procedures of FF collection are in Zachut et al. [1].

### 2.2 Sample preparation for proteomic analysis

Thirteen cows (7 CTL and 6 LFC) were subjected to follicular aspiration and in 12 cows (6 from each group), E_2_-active follicles were found. Two of these cows (1 CTL and 1 LFC) had 2 E_2_-active follicles, and as we preferred to examine cows with 1 dominant follicle, these animals were excluded from further analysis. In total, the characteristics of the preovulatory (E_2_-active) follicles and proteomic analysis were performed for follicles from 10 cows, 5 CTL cows and 5 LFC. Each FF sample was subjected to buffer exchange with 50 mM ammonium bicarbonate using a 3-kDa molecular weight cutoff filter (Amicon, Millipore, USA). Protein concentration from each sample was determined by its reading at 280 nm in a NanoDrop spectrophotometer (Thermo Scientific, USA) and comparison to a calibration curve using bovine serum albumin in 50 mM ammonium bicarbonate. Each sample contained 100 µg total protein. Proteins were denatured using 8 M urea and incubated for 10 min at room temperature followed by addition of 5 mM dithiothreitol and incubation for 1 h at room temperature. Urea was diluted five times and proteins were alkylated with 10 mM iodoacetamide (Sigma, USA) in the dark for 30 min at 21 °C. Proteins were then subjected to digestion with trypsin (Promega, Madison, WI, USA) at a 1:50 trypsin-to-protein ratio for 16 h at 37 °C [3]. The digestions were stopped by trifluroacetic acid (1% v/v) Samples were desalted using solid-phase extraction columns (Oasis HLB, Waters, Milford, MA, USA) and stored at −80 °C until further analysis.

### 2.3 Liquid chromatography

Ultra performance liquid chromatography (ULC) grade solvents were used for all chromatographic steps. Protein (2 µg) from each sample was loaded using split-less nano-ULC (10 kpsi nanoAcquity, Waters). The mobile phase consisted of: (A) H_2_O+0.1% formic acid and (B) acetonitrile+0.1% formic acid. Desalting of the samples was performed online using a reversed-phase C18 trapping column (180 µm internal diameter, 20 mm length, 5 µm particle size; Waters). The peptides were then separated using a an HSS T3 nano-column (75 µm internal diameter, 250 mm length, 1.8 µm particle size; Waters) at 0.35 µL/min. Peptides were eluted from the column using the following gradient: 4–35% B in 150 min, 35–90% B in 5 min, maintained at 90% for 5 min and then back to initial conditions.

### 2.4 MS

The nano-ULC was coupled online through a nano-ESI emitter (10 μm tip; New Objective, Woburn, MA, USA) to a quadrupole orbitrap MS (Q Exactive Plus, Thermo Scientific, USA) using a Flex Ion nano-spray apparatus (Proxeon). Data were acquired in data dependent acquisition (DDA) mode using a Top20 method. MS1 resolution was set to 60,000 (at 400 *m*/*z*) and maximum injection time was set to 20 ms. MS2 resolution was set to 17,500 and a maximum injection time of 60 ms.

### 2.5 Data processing and analysis

Differential expression was quantified by MS1 intensity-based label-free quantification [3]. Raw data were imported into Expressionist® software (Genedata) for processing as described previously [3]. The software was used for retention-time alignment and peak detection of precursor peptides. A master peak list was generated from all MS/MS events and sent for database target-decoy searching using Mascot v2.5.1 (Matrix Sciences). Data were searched against the bovine sequences UniprotKB version 2015_07 (http://www.uniprot.org/) appended with 125 common laboratory-contaminant proteins. Fixed modification was set to carbamidomethylation of cysteines and variable modifications included oxidation of methionines and deamidation of N or Q. Precursor mass tolerance was set to 10 ppm and product ion tolerance to 20 ppm. Search results were then filtered using the PeptideProphet [4] algorithm embedded in Scaffold (Proteome Software) to achieve a maximum false discovery rate of 1% at the protein level. Peptide identifications were imported back to expressions to annotate identified peaks. Proteins were quantified from the peptide data using an in-house script [3].

Data were normalized based on total ion current. Protein abundance was obtained by summing the 3 most intense, unique peptides per protein [5], unless the protein was identified by 2 or 1 peptide, in which case the quantification was based on only those peptides.

### 2.6 Statistical analysis

For proteomic data, Student׳s *t*-test, after logarithmic transformation, was used to identify significant differences across the biological replica. Fold changes were calculated based on the ratio of arithmetic means of the case versus control samples.
