# Supplementary material for: Proteome dataset of pre-ovulatory follicular fluids from less fertile dairy cows
Source: Data Brief. 2016 Apr 26;7:1515–8. doi: 10.1016/j.dib.2016.04.051 (PMC4857218; doi:10.1016/j.dib.2016.04.051)
Supplement: Supplementary file 2 — Supplementary material [file mmc2.docx]

**Conflict of interest**

The authors whose names are listed immediately below certify that they have NO affiliations with or involvement in any organization or entity with any financial interest (such as honoraria; educational grants; participation in speakers’ bureaus; membership, employment, consultancies, stock ownership, or other equity interest; and expert testimony or patent-licensing arrangements), or non-financial interest (such as personal or professional relationships, affiliations, knowledge or beliefs) in the subject matter or materials discussed in this manuscript.

Maya Zachut

Pankaj Sood

Lilya Livshitz

Gitit Kra

Yishai Levin

Uzi Moallem
